# Full-Endoscopic Transforaminal Ventral Decompression for Symptomatic Thoracic Disc Herniation with or without Calcification: Technical Notes and Case Series

**DOI:** 10.1155/2021/6454760

**Published:** 2021-11-03

**Authors:** Shangju Gao, Jingchao Wei, Wenyi Li, Long Zhang, Can Cao, Jinshuai Zhai, Bo Gao

**Affiliations:** Spinal Surgery Medical Team of Orthopedics, Hebei General Hospital, 348#Hepingxi Road, Shijiazhuang, Hebei, China

## Abstract

**Background:**

Symptomatic thoracic disc herniation is a challenge in spinal surgery, especially for cases with calcification. Traditional open operation has a high complication rate. The authors introduced a modified full-endoscopic transforaminal ventral decompression technique in this study and evaluated its imaging and clinical outcomes.

**Materials and Methods:**

Eleven patients with symptomatic thoracic disc herniation who underwent full-endoscopic transforaminal ventral decompression in a single medical center were enrolled. The surgical technique was performed as described in detail. Dilator sliding punching, endoscope-monitored foraminoplasty, and base cutting through the “safe triangle zone” are the key points of the technique. Clinical outcomes were assessed by the modified Japanese Orthopedic Association (mJOA) score for neurological improvement and the visual analogy score (VAS) for thoracic and leg pain. The operation time, hospital stay, and complications were also analyzed.

**Results:**

Postoperative magnetic resonance imaging (MRI) revealed good decompression of the spinal cord. The mJOA improved from 7.4 (range: 5–10) to 10.2 (range: 9–11). Axial thoracic pain improved in 8 of 9 patients. Leg pain and thoracic radicular pain improved in all patients. No complications were observed. The average operation time was 136 minutes (range: 70–180 minutes). The average length of hospital stay was 5.3 days (range: 2–8 days).

**Conclusion:**

Minimally invasive full-endoscopic transforaminal ventral decompression for the treatment of symptomatic thoracic disc herniation with or without calcification is feasible and may be another option for this challenging spine disease.

## 1. Introduction

Symptomatic thoracic disc herniation (TDH) is a relatively uncommon entity, constituting less than 1% of all disc herniations [1]. TDHs often occur in the 4–6^th^ decade of life and may present with radiculopathy and/or myelopathy. The symptoms range from slight back pain to severe intercostal neuralgia, weakness of the lower extremities, and even bowel and urine abnormalities [2, 3]. Surgery is reserved for those patients who are nonresponsive to conservative treatment.

Surgical treatment of TDHs is quite challenging. A variety of surgical approaches have been developed to treat TDHs, including transfacet pedicle-sparing [4], transpedicular [5], costotransversectomy [6], and lateral extracavitary [7]. However, each of these approaches has its own disadvantages and complications [8].

More recently, percutaneous transforaminal endoscopic discectomy (PTED) has been developed and achieved excellent clinical outcomes in the treatment of not only soft lumbar disc herniations (LDH) [9, 10] but also of calcified type of lumbar disc herniation [11–13]. However, there are few reports about endoscopic transforaminal thoracic discectomy to treat TDH.

In the present study, we introduced a posterolateral full-endoscopic transforaminal ventral decompression technique for thoracic disc herniation with or without calcification. This surgical technique is a modification of PTED for lumbar disc disease. We used this modified PTED technique to treat thoracic disc herniation and achieved good clinical outcomes.

## 2. Materials and Methods

This study was approved by the Ethics Committee of the Hebei General Hospital before data collection and analysis. It was a retrospective study. The diameter of the endoscope system was 4.3 mm, with a view angle of 30° and 181 mm working length through a working channel with an inside diameter of 6.9 mm (SPINENDOS GmbH, Munich, Germany).

### 2.1. Clinical Data

Between January 2018 and December 2019, eleven patients with thoracic disc herniation underwent full-endoscopic transforaminal ventral decompression in a single medical center. Seven were male and four were female, aged 25–72 years (mean: 53 years). Patients with a history of fracture, infection, or tumors were excluded. Pathological changes were only or mainly located in the ventral side of the spinal cord, which was clinically recognized as the main cause of the symptoms. As confirmed by the MRI and CT scan, the pathological changes included soft disc herniation (2 cases) and calcified disc herniation (9 cases). Two was located at T9-T10, 3 at T10-T11, 5 at T11-T12, and 1 at T12-L1. The presenting symptoms were classified as axial thoracic pain, thoracic radicular pain, leg pain, and myelopathy (6). The mean symptom duration was 8.6 months (range 2–18 months). The clinical characteristics of the patients are given in Table 1.

### 2.2. Surgical Technique

#### 2.2.1. Preoperative Preparation and Anesthesia

CT, MRI, and X-ray examinations were performed before the operation. The patients needed to simulate the operation position under the care of the medical staff. There were two advantages: first, the patients could adapt to the position of the operation in advance to avoid anxiety during the operation. Second, whether there were neurological deficits in a specific position could be identified preoperatively. For the operation, the patient was positioned in a comfortable prone position. Some soft cushions were used to keep the patients in a comfortable position and relieve their neurological symptoms. The anesthesia regime was local anesthesia combined with conscious sedation. During this, an electrocardiograph, blood pressure, respiration, and finger pulse oxygen saturation were monitored. Dexmedetomidine hydrochloride was pumped at a rate of 0.1–0.5 *μ*g/kg/hour. The speed of the pump was adjusted according to the patient's surgical tolerance, so that the patient was maintained in a sober yet sedated state. Anesthesiologists took care of the patients throughout the operation. The local anesthetic was a mixture of 0.5% lidocaine and 0.25% ropivacaine. The anesthesia area included the skin, deep fascia, dorsolateral articular process, and intervertebral foramen. No anesthetic was given directly into the spinal canal. This can prevent the spinal cord from being anesthetized, resulting in serious complications.

#### 2.2.2. Working Port Establishment

**Figure 1:**
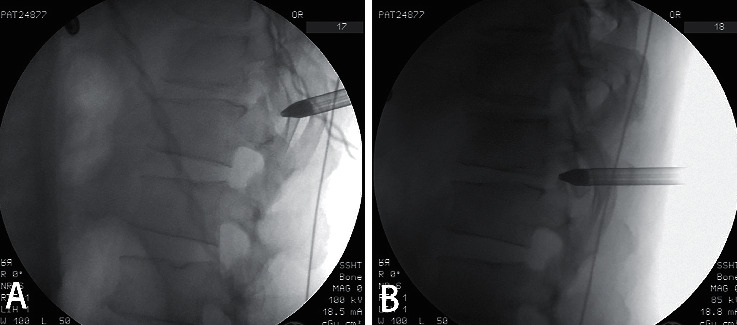
Images of X-ray fluoroscopy showing the dilator punching procedure: a dilator with blunt tip located on the lateral side of the articular process (a). The dilator slid into the lower part of the intervertebral foramen (b).

**Figure 2:**
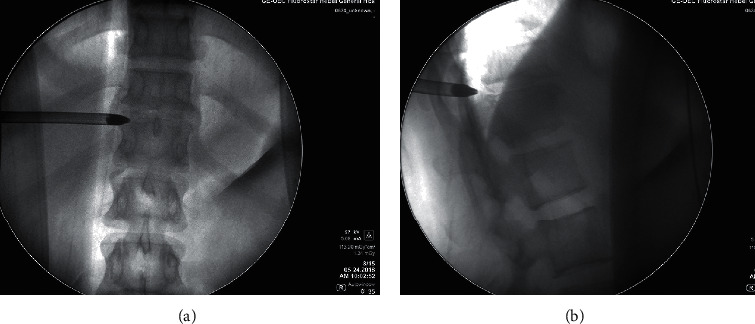
The dilator is located at the lower part of the intervertebral foramen, as can be checked on the lateral X-ray fluoroscopy (a) and anteroposterior X-ray fluoroscopy (b).

First, we located the puncture trajectory under X-ray fluoroscopy. The puncture trajectory was consistent with the direction of the intervertebral space. The entry point was located on the medial side of the highest point of the posterior rib. The distance to the spinous process was approximately 6–8 cm. A 16G spinal needle was used for the anesthesia of the skin and the deep fascia. After making an incision, a hollow dilator with a diameter of 6.5 mm was used for the following puncture process. A dilator with a blunt tip could reduce the risk of pleura and spinal cord injury. The punching target was the lateral side of the articular process. The pinhole in the dilator can be used to inject anesthetics along the puncture path. After AP and lateral view were checked by X-ray, the dilator was slid into the lower part of the intervertebral foramen (Figures 1 and 2). The working sheath could be introduced along the dilator. Foraminoplasty and spinal cord decompression were performed subsequently under the endoscopic view.

#### 2.2.3. Endoscopic Operation

**Figure 3:**
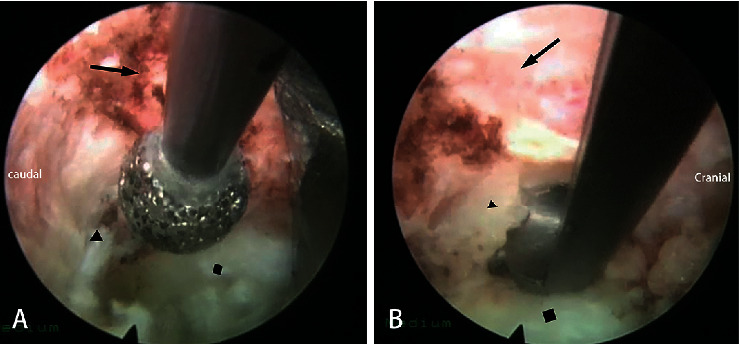
Endoscopic foraminoplasty by the high-speed diamond burr (a) and Kerrison rongeur (b). 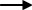 The articular process, 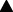 the ligamentum flavum, and the 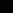 thoracic disc.

After the soft tissue in the foramen was removed, the bony anatomical structure of the intervertebral foramen could be clearly exposed to view. Foraminoplasty was performed with a high-speed diamond burr or a Kerrison rongeur (Figure 3). In the foraminoplasty, the excised bony structure included not only the ventral side of the superior articular process but also part of the posterior edge of the vertebral body, even the upper part of the pedicle. The key point of the foramen foraminoplasty is to increase the range of motion of the working channel. This modified foraminoplasty could not only preserve the stable structure of the spine as much as possible but also expose the ventral pathology clearly. When the space of the intervertebral foramen area was ample enough, the working sheath could be further deepened. There was no extra pressure on the spinal canal during this process. Forced entry of the working sheath was forbidden.

**Figure 4:**
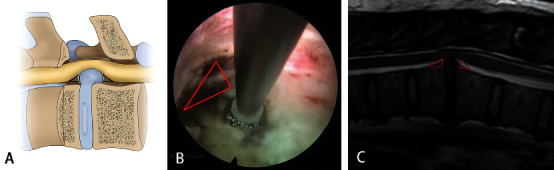
The “safe triangle zone” described by a diagram (a), endoscopic view (b), and MRI.

**Figure 5:**
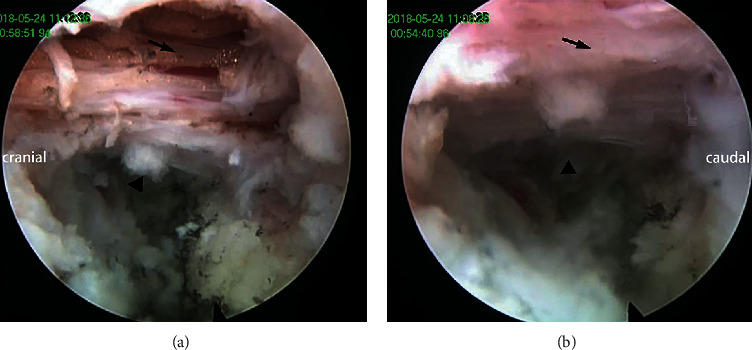
The decompressed central spinal canal (a) and lateral spinal canal under endoscopic view (b) 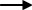 showing the spinal cord and 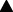 intervertebral space.

After entering the lateral spinal canal, for a soft disc herniation, discectomy was performed by the “out-in-out” technique. The fibrous ring was exposed and cut open. The soft nucleus pulposus in the intervertebral space could be cleaned. When the space was large enough, the endoscope and surgical instruments would be inserted. The disc herniated into the spinal canal could be seen at 12 o'clock in the field of vision. Discectomy can be achieved by the 30-degree visual field angle of the endoscope and variable angle forceps. Central spinal canal decompression can be achieved by a broad foraminoplasty and working channel pressed down. However, this technique was only suitable for soft lesions. In some cases, the pathogenic factor was a calcified disc, osteophytes, or both. First, the soft disc was removed as described, and a tough shell remained. Bony decompression began at the cranial and caudal edge of the vertebrae, which was the base of the hypertrophic fibrous ring and osteophyte. We can see the spinal cord was jacked up at the distal side of the calcification. Epidural fat is filled in this area. After exposure and hemostasis, we could say a space between the spinal cord and calcification. The space was safe for manipulation, and it was named the “safe triangle zone” by our team (Figure 4). The three sides of the triangle were calcification, spinal cord, and posterior wall of the vertebral body. The tools could operate in this area without touching the spinal cord. After the base was disrupted by the high-speed diamond burr, the hump could be cut off piece by piece. Whether the posterior longitudinal ligament needed to be removed was dependent on the rate of dural sac relaxation. Adequate decompression should be the first aim of the surgery (Figure 5). The operation is over after careful hemostasis. No drainage system was applied. Patient feedback is essential throughout the entire operation.

#### 2.2.4. Postoperative Outcome Evaluation

**Figure 6:**
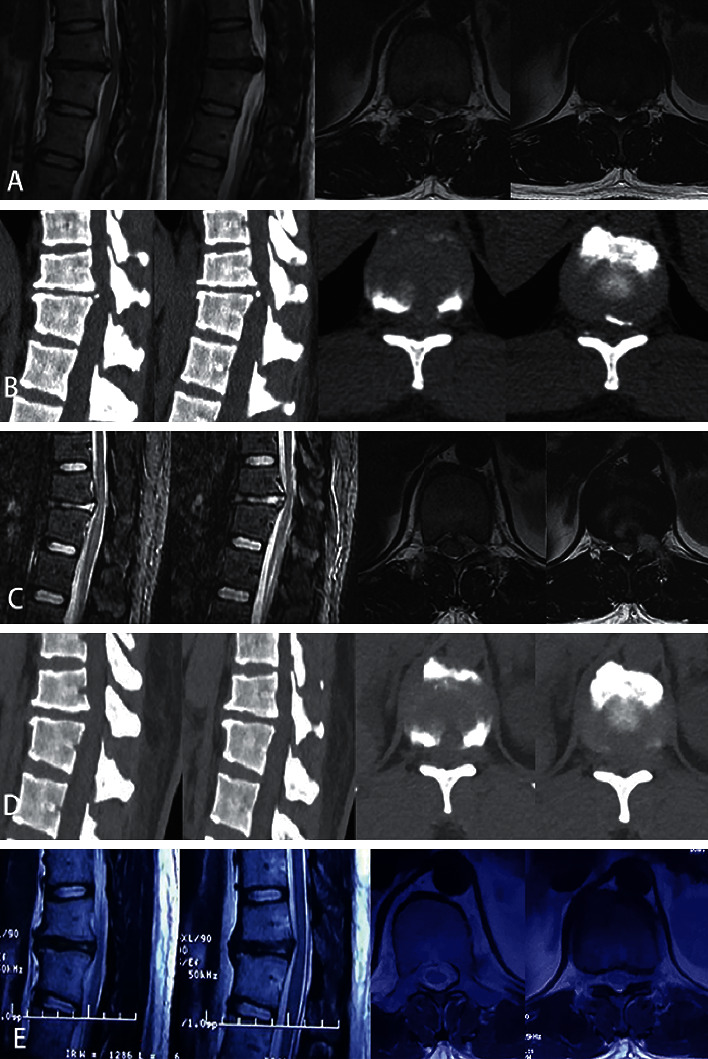
Preoperative MRI (a) and CT (b) scan showing spinal cord compression by calcified disc herniation and posterior vertebral osteophyte at T11-T12 (case 3). Postoperative MRI (c) and CT scan (d) showing sufficient decompression of the spinal cord and the calcified disc was cut off clearly. MRI at the 3-month follow-up (e) showing the spinal cord surrounded by cerebrospinal fluid.

MRI was used as the radiological assessment, which was performed within 2 days after the operation as well as during the follow-up if necessary (Figure 6). The neurological outcome was assessed using the modified Japanese Orthopedic Association (mJOA) score (11 points) [14]. The degree of axial/radicular thoracic and leg pain was assessed using the visual analogy score (VAS). The operation time and the length of hospital stay were also recorded. The operation time was defined as the time from puncture to suturing. It did not include the time of the operative positioning or the operation target locating. The hospital stay for rehabilitation and physical therapy for patients with myelopathy was not reckoned in the length of hospital stay.

## 3. Results

All patients in this study successfully underwent surgery as described. Follow-up was available for all of the patients, and it ranged from 13 months to 24 months (average: 15 months). Postoperative MRI imaging revealed good decompression of the spinal cord in all 11 patients.

Intraoperative blood loss was not measured due to continuous fluid irrigation. However, no patient required any intra or postoperative blood transfusion. There were no serious complications such as nerve injury, infection, or hematoma. The average operation time was 136 minutes (range: 70–180 minutes). The average length of hospital stay was 5.3 days (range: 2–8 days). The mJOA improved from 7.4 (range 5–10) preoperatively to 10.2 (range 9–11) at the last follow-up. Axial thoracic pain improved in 8 of 9 patients with this symptom. Leg pain and thoracic radicular pain improved in all of the patients with this symptom (Table 2).

## 4. Discussion

Because of the underlying anatomy, ventral decompression of the thoracic spinal canal is a technical challenge. Open decompression inflicts great trauma, has a high rate of complications, and possibly requires additional internal fixation [4, 7, 8]. With the improvement of minimally invasive surgery, video-assisted thoracoscopic surgery, microendoscopic surgery, and full-endoscopic surgery have achieved satisfactory clinical outcomes and fewer complications in the treatment of TDH [15–17]. In this retrospective clinical study, we introduced a modified full-endoscopic ventral decompression technique for TDH. The imaging and clinical results were satisfactory. Postoperative MRI imaging in all cases showed sufficient decompression of the spinal cord. MRI also showed the presence of cerebrospinal fluid between the spinal cord and the dura. mJOA and VAS were improved in all cases except 1 case of axial thoracic pain. Similar surgical techniques and findings have been presented in some previous reports [18–22].

We have made some modifications to the previously reported transforaminal endoscopic ventral decompression techniques. In the punching procedure, we used a dilator downward sliding technology instead of the needle targeting puncture technique to avoid neurovascular injury and pleural injury. Pulmonary complications are one of the troublesome complications of thoracic spine surgeries. Entering the thoracic cavity is the immediate cause, which is more likely to occur in the anterior approach [23, 24]. Although no such complication has been reported for transforaminal endoscopic surgery, we believe that this potential risk exists.

In the foraminoplasty procedure, we used a high-speed diamond burr and a Kerrison rongeur under an endoscopic view instead of a circular saw or bone drill under fluoroscopy to avoid spinal cord injury and to preserve the stability of the spinal posterior column as much as possible. Hua et al. applied this similar technique in lumbar surgery. The neurological complication rate was 1.4%, which is much lower than for traditional PTED surgery (12.4%) [25]. In the establishment of the working channel procedure, we used the “step-by-step” technique instead of the “one-step” technique [19, 21] to avoid any iatrogenic pressure on the spinal canal during the working channel insertion. Wagner et al. applied this working channel establishing technique to a 31-year-old female with T8-9 disc herniation. The imaging and clinical outcomes were excellent [20]. We also reported the sequence applied for endoscopic decompression. Rutten et al. reported a similar technique called “box-shaped” [26]. However, there are some differences. First, for central calcified discs, it is difficult to resect the base directly with the burr because of the obstruction of the spinal cord. A strong pair of forceps needs to reach the safe triangle zone to complete the bone resection. Second, clear exposure of the safe triangle zone before bone resection can help us accurately judge the boundary of resection and the distance from the spinal cord. As far as we know, this is the first time anyone has described the concept of the “safe triangle zone.”

The clinical outcomes in this trial are similar to those in previous reports. Guo et al. reported 6 cases of symptomatic TDH. The mJOA improved from 4.4 preoperatively to 6.6 one year after surgery [19]. Choi et al. presented a mean VAS improvement from 6.5 to 3.0 for back pain and 5.8 to 2.5 for leg pain. However, only patients with soft disc herniations were included [27]. Rutten et al. reported a 20% (5/25) rate of complications including 1 dural tear, 1 epidural hematoma, 2 transient intercostal neuralgias, and 1 deterioration of myelopathy [28]. In our previous research on the endoscopic surgery for thoracic OLF, we found complications such as neck pain and dural tear [29]. However, no complication was observed in this study or in Choi and Guo's. This may be due to the small sample size.

Because of the low incidence rate of TDH, this study is limited by its small sample size. It is not sufficient to show that this method is a safe and effective surgical technique. Larger controlled studies are warranted. Long-term follow-up and analysis will be required in the future.

## 5. Conclusion

Full-endoscopic transforaminal ventral decompression for the treatment of symptomatic thoracic disc herniation with or without calcification is feasible and may be another option for this challenging spine disease.

## Figures and Tables

**Table 1:** Summary of the clinical and imaging characteristics of the 8 cases who underwent full-endoscopic transforaminal ventral decompression surgery.

No. of patients	Gender	Age (years)	Location	Primary symptoms and duration (months)	Soft/calcified
1	Male	59	T11-T12	Axial thoracic pain (12), thoracic radicular pain (7)	Soft
2	Male	65	T10-T11	Axial thoracic pain (6), myelopathy (4)	Calcified
3	Female	25	T11-T12	Axial thoracic pain (12), thoracic radicular pain (7)	Calcified
4	Female	72	T9-T10	Thoracic radicular pain (8), myelopathy (8)	Calcified
5	Male	28	T11-T12	Leg pain (6), myelopathy (3)	Calcified
6	Female	63	T11-T12	Axial thoracic pain (18), leg pain (4), myelopathy (2)	Calcified
7	Male	39	T12-L1	Myelopathy (2)	Calcified
8	Male	55	T10-T11	Axial thoracic pain (9), leg pain (2), myelopathy (2)	Calcified
9	Male	68	T11-T12	Axial thoracic pain (14), myelopathy (3)	Calcified
10	Female	71	T9-T10	Axial thoracic pain (6), thoracic radicular pain (1)	Calcified
11	Male	42	T10-T11	Myelopathy (2)	Soft

**Table 2:** Operative data, length of hospital stay, and pre and postoperative mJOA and VAS assessed at the last follow-up.

No. of patients	Operation time (mins)	Hospital stay (days)	mJOA		Axial VAS		Leg or thoracic radicular VAS	
			Pre-op	Post-op	Pre-op	Post-op	Pre-op	Post-op
1	70	7	10	11	4	1	6	0
2	160	5	6	10	3	1	0	0
3	120	7	9	11	3	3	6	2
4	95	7	6	9	0	2	3	0
5	180	8	8	10	0	0	5	0
6	180	8	5	9	2	2	3	1
7	80	4	7	9	0	0	0	0
8	170	4	9	11	3	2	2	0
9	160	3	6	10	4	1	3	2
10	130	2	8	11	2	0	3	0
11	150	3	7	11	2	0	3	0

## Data Availability

The data generated or analyzed during this study are included in the article.

## Abbreviations

mJOA: Modified Japanese Orthopedic Association
MRI: Magnetic resonance imaging
VAS: Visual analogue scale
LDH: Lumbar disc herniation
OLF: Ossification of the ligamentum flavum
PTED: Percutaneous transforaminal endoscopic lumbar discectomy.

## References

[1] Yoshihara H. (2014). Surgical treatment for thoracic disc herniation: an update. *Spine*.

[2] Dimar J. R., Bratcher K. R., Glassman S. D., Howard J. M., Carreon L. Y. (2008). Identification and surgical treatment of primary thoracic spinal stenosis. *American Journal of Orthopedics (Belle Mead, N.J.)*.

[3] Hur J.-W., Kim J.-S., Seung J.-H. (2019). Full-endoscopic interlaminar discectomy for the treatment of a dorsal migrated thoracic disc herniati on: case report. *Medicine*.

[4] Bransford R., Zhang F., Bellabarba C., Konodi M., Chapman J. R. (2010). Early experience treating thoracic disc herniations using a modified transfacet pedicle-sparing decompression and fusion. *Journal of Neurosurgery: Spine*.

[5] Le Roux P. D., Haglund M. M., Harris A. B. (1993). Thoracic disc disease: experience with the transpedicular approach in twenty consecutive patients. *Neurosurgery*.

[6] Simpson J. M., Silveri C. P., Simeone F. A., Balderston R. A., An H. S. (1993). Thoracic disc herniation. re-evaluation of the posterior approach using a modified costotransversectomy. *Spine*.

[7] Maiman D. J., Larson S. J., Luck E., El-Ghatit A. (1984). Lateral extracavitary approach tothe spine for thoracic disc herniation: report of 23 cases. *Neurosurgery*.

[8] Brotis A. G., Tasiou A., Paterakis K., Tzerefos C., Fountas K. N. (2019). Complications associated with surgery for thoracic disc herniation: a systematic review and network meta-analysis. *World Neurosurgery*.

[9] Xu J., Li Y., Wang B. (2019). Percutaneous endoscopic lumbar discectomy for lumbar disc herniation with modic changes via a transforaminal approach: a retrospective study. *Pain Physician*.

[10] Nakamae T., Fujimoto Y., Yamada K. (2019). Transforaminal percutaneous endoscopic discectomy for lumbar disc herniation in athletes under the local anesthesia. *Journal of Orthopaedic Science*.

[11] Chen Y., Wang J.-X., Sun B. (2019). Percutaneous endoscopic lumbar discectomy in treating calcified lumbar intervertebral disc herniation. *World Neurosurgery*.

[12] Kim H. S., Paudel B., Jang J. S., Lee K., Oh S. H., Jang I. T. (2018). Percutaneous endoscopic lumbar discectomy for all types of lumbar disc herniations (LDH) including severely difficult and extremely difficult LDH cases. *Pain Physician*.

[13] Dabo X., Ziqiang C., Yinchuan Z. (2016). The clinical results of percutaneous endoscopic interlaminar discectomy (PEID) in the treatment of calcified lumbar disc herniation: a case-control study. *Pain Physician*.

[14] Zhang J., Wang L., Li J., Yang P., Shen Y. (2016). Predictors of surgical outcome in thoracic ossification of the ligamentum flavum: focusing on the quantitative signal intensity. *Scientific Reports*.

[15] Yüce İ., Kahyaoğlu O., Çavuşoğlu H. A., Çavuşoğlu H., Aydın Y. (2018). Midterm outcome of thoracic disc herniations that were treated by microdiscectomy with bilateral decompression via unilateral approach. *Journal of Clinical Neuroscience*.

[16] Bordon G., Burguet Girona S. (2017). Experience in the treatment of thoracic herniated disc using image-guided thorascopy. *Revista Española de Cirugía Ortopédica y Traumatología*.

[17] Bydon M., Gokaslan Z. (2014). Minimally invasive approaches in the treatment of thoracic disk herniation. *World Neurosurgery*.

[18] Liu W., Yao L., Li X. (2019). Percutaneous endoscopic thoracic discectomy via posterolateral approach: a case report of migrated thoracic disc herniation. *Medicine*.

[19] Guo C., Zhu D., Kong Q. (2019). Transforaminal percutaneous endoscopic decompression for lower thoracic spinal stenosis. *World Neurosurgery*.

[20] Wagner R., Telfeian A. E., Iprenburg M. (2016). Transforaminal endoscopic foraminoplasty and discectomy for the treatment of a thoracic disc herniation. *World Neurosurgery*.

[21] Xiaobing Z., Xingchen L., Honggang Z. (2019). “U” route transforaminal percutaneous endoscopic thoracic discectomy as a new treatment for thoracic spinal stenosis. *International Orthopaedics*.

[22] Paolini S., Tola S., Missori P., Esposito V., Cantore G. (2016). Endoscope-assisted resection of calcified thoracic disc herniations. *European Spine Journal*.

[23] Hurley E. T., Maye A. B., Timlin M., Lyons F. G. (2017). Anterior versus posterior thoracic discectomy: a systematic review. *Spine*.

[24] Yoshihara H., Yoneoka D. (2014). Comparison of in-hospital morbidity and mortality rates between anterior and nonanterior approach procedures for thoracic disc herniation. *Spine*.

[25] Hua W., Zhang Y., Wu X. (2019). Full-endoscopic visualized foraminoplasty and discectomy under general anesthesia in the treatment of L4-L5 and L5-S1 disc herniation. *Spine*.

[26] Ruetten S., Hahn P., Oezdemir S., Baraliakos X., Godolias G., Komp M. (2018). Operation of soft or calcified thoracic disc herniations in the full-endoscopic uniportal extraforaminal technique. *Pain Physician*.

[27] Choi K. Y., Eun S. S., Lee S. H., Lee H. Y. (2010). Percutaneous endoscopic thoracic discectomy; transforaminal approach. *Minimally Invasive Neurosurgery*.

[28] Ruetten S., Hahn P., Oezdemir S. (2018). Full-endoscopic uniportal decompression in disc herniations and stenosis of the thoracic spine using the interlaminar, extraforaminal, or transthoracic retropleural approach. *Journal of Neurosurgery: Spine*.

[29] Li W., Gao S., Zhang L., Cao C., Wei J. (2020). Full-endoscopic decompression for thoracic ossification of ligamentum flavum: surgical techniques and clinical outcomes: a retrospective clinical study. *Medicine*.

